# Is ethyl chloride the new nitrous oxide? A case report

**DOI:** 10.1186/s12883-024-03689-x

**Published:** 2024-06-04

**Authors:** Quentin Salardaine, Clément Desjardins, Guillaume Baille, Emmanuel Roze, Clotilde Nardin

**Affiliations:** 1 Neurology Department, Saint-Denis Hospital, Saint-Denis, France; 2 Neurology Department, Fondation Adolphe de Rothschild, Paris, France; 3 Sorbonne University, APHP—Salpêtrière Hospital, CNRS, INSERM, Paris Brain Institute, Paris, France

**Keywords:** Ethyl chloride, Cerebellar, Intoxication, Neurotoxicity, Case report

## Abstract

**Background:**

Over the last decade, there has been an emerging trend of recreational misuse of several drugs and inhaled solvent including ethyl chloride. This case report follows CARE guidelines and highlights, with supporting video, the neurological features of ethyl chloride intoxication.

**Case presentation:**

A 48-year-old man was seen for the sudden occurrence of an unsteady gait with dizziness. His only medical history was a chronic and treated HIV infection without any complications. Clinical examination revealed a cerebellar syndrome associated with impairment of short-term memory. Biological and radiological workups were normal. After several days, the patient recalled ethyl chloride inhalation. He fully recovered after being discharged from hospital.

**Conclusion:**

Clinicians should recognise the clinical features and neurological manifestations of ethyl chloride intoxication due to the potential fatal cardiovascular complications of this intoxication.

**Supplementary Information:**

The online version contains supplementary material available at 10.1186/s12883-024-03689-x.

 Ethyl chloride (EC) (C_2_H_5_Cl) is a volatile gas mainly used as topical anaesthetic due to its refrigerant properties [1]. In the context of chemsex, i.e., sexual activity while under the influence of drugs, there has been an emerging trend of recreational misuse of several drugs (e.g. methamphetamine, gamma-hydroxybutyrate and mephedrone) and inhaled solvent including EC [1, 2]. The current knowledge about the consequences of EC intoxication is mostly based on reports of single cases [3, 4]. Cardiovascular events are the most frequent complication whereas neurological damage is thought to be rare. Here, we report a detailed case of neurotoxicity due to EC intoxication.

## Case report

A 48-year-old man was seen for the sudden occurrence of an unsteady gait with dizziness. He had an HIV infection treated by dolutegravir and rilpivirine for about 18 years without any history of opportunistic infections. After two days without answering to friends’ calls, he was found asleep at home by emergency services, unable to walk, with slurred speech, and admitted to our centre for investigations. On admission, he had a static cerebellar syndrome with ataxic gait, unsteady tandem gait, ataxic dysarthria and nausea associated with impairment of short-term memory with fluctuating attention (See video two days after admission). Otherwise, the examination was normal, with no observed nystagmus or tremors). Blood count and metabolic investigations, including vitamins dosage, glycemia, ionogram, calcemia, ammonia, and creatinine, were unremarkable. CD4 count was normal and HIV was not detected in blood. Urine drug screening was negative. Infectious and auto-immune investigations were normal. Cerebrospinal fluid (CSF) analysis was normal (2 WBC/mm3, 0 RBC/mm3, protein 0.37 g/L) with no detection of JC virus or HIV. Brain magnetic resonance imaging (MRI) and electrocardiogram were normal. The condition of the patient gradually improved without any treatment so that he was able to walk unaided three days after admission (with only mild ataxia). He eventually recalled misusing (inhalation) an EC spray two days before admission. He had already been inhaling EC occasionally, in smaller quantities in order to relax himself before sleep or to alleviate muscular pain due to sport. This time, he estimated his acute inhaled consumption at two full sprays of 175 mL of EC each. He fully recovered two weeks after being discharged from hospital. Based on the medical history and clinical observation, we retained the diagnosis of acute ataxia related to EC intoxication.

## Discussion

Acute-onset ataxia should prompt physicians to first consider the possibility of a cerebellar stroke. Here, normal MRI together with memory impairment and initial sleepiness were rather suggestive of an alternative cause, especially drug or toxic exposure. In our case, the diagnosis was further delayed because (i) EC is not routinely detected in urine (ii) neurological complications of EC are poorly known (iii) the patient had memory disturbances initially preventing him to mention EC use. EC is commonly used for local muscle anaesthesia in sportive or traumatic contexts [3]. Several sprays containing EC are easily and legally available over the counter or through e-commerce companies. The most frequent manifestations related to EC misuse are cardiovascular symptoms such as arrhythmia and gastrointestinal problems [3]. Due to its lipophilic properties, EC easily passes through the blood brain barrier and is thought to inhibit N-methyl-D-aspartate (NMDA) receptors [5]. EC intoxication causes central nervous system depression, sensation of drunkenness and hallucinations [3].

Even though the recreational use of EC has been reported since the 1980s, the literature is scarce and only a few cases of intoxication are reported [3, 4]. Of the twelve reported cases (including ours), three had HIV infection and intoxication could occur after a one-time use or after several years of repeated use [3] (Table 1). Reported neurological effects comprised cognitive and psychiatric symptoms (euphoria, hallucinations, short-term memory impairment, confusion), cerebellar syndrome, and pyramidal syndrome (Table 1). These manifestations usually resolve within days to weeks with supportive care [3]. However, several deaths have been associated with EC use, mostly due to cardiac complications [6]. In our patient, the desired effect was drowsiness state. He had already inhaled EC before – though in small amounts - without experiencing any cognitive or adverse motor effects. Anecdotally, on website forums for recreational drug, EC is described as a substitute for poppers or nitrous oxide, but with longer effect. The current tendency to misuse legal inhaled chemicals (such as nitrous oxide) for recreational purposes raises the possibility of a forthcoming increase in the number of patients with neurological manifestations due to EC intoxication. EC intoxication should thus be considered in patients with unexplained acute neurological manifestations.

**Table 1:** Review of previous case reports of neurotoxicity due to ethyl chloride

Author	Sex	Age (years)	Symptoms	Neurological signs	Duration of use	Recovery after EC cessation
Nordin et al. [7]	M	52	Confusion, hallucinations, **dizziness**	**Ataxia** (unsteady walk), **short-term memory impairment**	30 years	Full recovery within 3 weeks of cessation
Soult and Walker [8]	M	22	Vertigo, blurred vision, diplopia, hallucinations	Marked **ataxia** (unable to walk), end-point nystagmus	4 months	Full recovery within 1 week of cessation
Finch and Lobo [9]	M	41	Hallucinations, drowsiness, shakiness, **inability to walk**	**Ataxia**, **dysarthria**, tremors, general weakness	2 years	Full recovery within 1 week of cessation
Demarest et al. [10]	M	45	**Difficulty walking**, **unsteadiness**, **slurred speech**	**Wide-based gait**, ankle and patellar clonus, symmetric brisk deep tendon reflexes	5 months	Full recovery within 1 week of cessation
Senussi and Chalise [3]	M	47	**Dizziness**, **unsteadiness**, **slurred speech**.	**Ataxia**, **wide-based gait**, mild **dysarthria**, mild aphasia	Single use	Full recovery within 1 week of cessation
Al-Ajmi et al. [4]	F	40	**Unsteady gait**, **dizziness**, hallucinations.	**Dysarthria**, horizontal nystagmus, ankle clonus, kinetic and intention tremors, **ataxic wide-based gait**	5 months	Full recovery within 3 weeks of cessation
Kuthiah and Er [11]	M	24	**Nausea**, abdominal cramps, **unsteady gait**	Intention tremors, bilateral nystagmus on horizonal gaze	2 months	Full recovery within 2 weeks of cessation
Young et al. [12]	M	42	Altered mentation, **unable to walk without support**, hallucinations	Bilateral lower extremity weakness with paresthesia, **dysarthria**, tremors of all extremities	1,5 years	Partial recovery within 2 months of cessation. Persistent difficulties in walking
Winkler et al. [13]	M	36	**Inability to walk**, **slurred speech**, **inability to stand without support**	**Ataxia**, loss of toe-finger proprioception, horizontal and vertical nystagmus, dysmetria	15 years	Full recovery within 2 weeks of cessation
Demir et al. [14]	F	24	**Gait abnormality**, blurred vision, shaky hands	Mild confusion, **dysarthria**, gaze evoked nystagmus, **ataxia of the trunk and gait**.	2 months	Full recovery within few days of cessation

The symptoms also presented by our patient are highlighted in bold. Cases solely documenting sudden death, devoid of neurological symptoms, have been omitted

The symptoms also presented by our patient are highlighted in bold. Cases solely documenting sudden death, devoid of neurological symptoms, have been omitted.

### Supplementary Information

Supplementary Material 1: Video. Video illustrating the neurological phenotype of the patient, at day 2 of hospitalisation.

## Data Availability

No datasets were generated or analysed during the current study.

## Abbreviations

EC: Ethyl chloride
MRI: Magnetic resonance imaging
NMDA: N-methyl-D-aspartate

## Declarations

We confirm that we have read the Journal’s position on issues involved in ethical publication and affirm that this work is consistent with those guidelines.

E.R. received honorarium for speech from Orkyn, Aguettant, Elivie, Merz-Pharma and for participating in advisory boards from Merz-Pharma, Ipsen, Elivie, Teva, and BIAL. He received research support from Merz-Pharma, Orkyn, Aguettant, Elivie, Ipsen, Everpharma, Enjoysharing, Fondation Desmarest, AMADYS, ADCY5.org, Fonds de dotation Patrick Brou de Laurière, Agence Nationale de la Recherche, Dystonia Medical Research Foundation, Hope For Annabel, Cure Alternating Hemiplegia of Childhood Alternating Hemiplegia of Childhood Foundation, Alternating Hemiplegia of Childhood Association of Iceland Association française de l’hémiplégie alternante, Alternating Hemiplegia of Childhood Kids of Nederland.

Other authors report no conflicts or relevant disclosures for the previous 12 months.

## References

[1] Pothiawala S, Yong CK, Charles R (2021). Inhaling muscle spray: a rising trend of abuse. World J Crit Care Med.

[2] Moreno-Gámez L, Hernández-Huerta D, Lahera G (2022). Chemsex and psychosis: a systematic review. Behav Sci.

[3] Senussi MH, Chalise S (2015). Acute reversible neurologic deficits due to ethyl chloride sniffing: a case report and review of literature. Am J Ther.

[4] Al-Ajmi AM, Morad MA, Cooper PE, Hassino LH, Siddeiq MA (2018). Reversible ethyl chloride neurotoxicity: a case report. Can J Neurol Sci.

[5] Tormoehlen LM, Tekulve KJ, Nañagas KA (2014). Hydrocarbon toxicity: a review. Clin Toxicol.

[6] Hong IZ (2022). Death related to ethyl chloride inhalation abuse: a case report. World J Emerg Med.

[7] Nordin C, Rosenqvist M, Hollstedt C (1988). Sniffing of ethyl chloride-an uncommon form of abuse with serious mental and neurological symptoms. Int J Addict.

[8] Soult TA, Walker JS (1993). Ethyl chloride intoxication. Am J Emerg Med.

[9] Finch CK, Lobo BL (2005). Acute inhalant-induced neurotoxicity with delayed recovery. Ann Pharmacother.

[10] Demarest C, Torgovnick J, Sethi NK, Arsura E, Sethi PK (2011). Acute reversible neurotoxicity associated with inhalation of ethyl chloride: a case report. Clin Neurol Neurosurg.

[11] Kuthiah N, Er C (2019). “High” on muscle spray – ethyl chloride abuse. Ann Acad Med Singap.

[12] Young R, et al. Recognizing ethyl chloride neurotoxicity: inhalant abuse hidden in plain sight. Cureus. 2023. 10.7759/cureus.37795. 10.7759/cureus.37795 PMC10195209 37214062

[13] Winkler GA, Dilbarova R, Clark RF, Schneir A, Minns AB (2023). Reversible neurotoxicity due to excessive use of ethyl chloride. J Emerg Med.

[14] Demir M, Özdilek B, Mayda Domaç F, Ülker M, Kenangil G. Ataxia which develops due to freeze spray abuse: a case study. Addicta. 2015. 10.15805/addicta.2015.2.2.0001E.

